# Dramatic response to neoadjuvant savolitinib in marginally resectable lung adenocarcinoma with MET exon 14 skipping mutation: A case report and literature review

**DOI:** 10.3389/fonc.2022.1006634

**Published:** 2022-10-27

**Authors:** Jiangfang Tian, Zhen Lin, Yueyun Chen, Yang Fu, Zhenyu Ding

**Affiliations:** Department of Biotherapy, Cancer Center, West China Hospital, West China Medical School, State Key Laboratory of Biotherapy, Sichuan University, Chengdu, China

**Keywords:** neoadjuvant therapy, savolitinib, MET exon 14 skipping mutation (METex14), NSCLC, case report, major pathological response

## Abstract

Mesenchymal–epithelial transition (MET) exon 14 skipping mutation (METex14) is a low-frequency driver mutation in metastatic non-small cell lung cancer (NSCLC) (3%–4%) and is associated with a poor prognosis. With the advent of selective MET inhibitors such as capmatinib, tepotinib, and savolitinib, the outcome for these patients was significantly improved. Here, we report a 76-year-old male patient with marginally resectable stage IIIB lung adenocarcinoma harboring METex14 who was successfully treated with savolitinib for neoadjuvant therapy. An 82% shrinkage of the primary tumor was observed, and only 5% of the tumor was viable by pathology in the following radical surgery. A dozen of studies tested the efficiency of neoadjuvant immunotherapy or immunochemotherapy, but for NSCLC with driver mutations, neoadjuvant targeted therapy might be more appropriate. We advocated the neoadjuvant MET TKI treatment for NSCLC.

## Introduction

The mesenchymal–epithelial transition (MET) factor, as a receptor for the hepatocyte growth factor (HGF), is encoded by the MET gene and plays a vital role in cancer progression. The major MET alterations were MET amplification and overexpression and MET exon 14 skipping mutation (METex14) (1). Specifically, METex14 occurs in approximately 3%–4% of non-small cell lung cancer (NSCLC) cases and is associated with a worse prognosis, which has been a novel treatment target for NSCLC (2–5). In China, savolitinib was approved for the treatment of locally advanced or metastatic NSCLC with METex14 in patients who have progressed after or who are intolerant to platinum-based chemotherapy in 2021 (2).

To improve the prognosis of lung cancer, neoadjuvant strategies for NSCLC have aroused great interest. The significance of a molecular targeted agent as a preoperative treatment is currently unknown, whereas immunotherapy (IO) has shown promising results in phase 2 or 3 studies such as the LCMC3 (6), CheckMate-816 (7), and NADIM (8). It was observed that subjects with sensitive driver mutations usually were excluded from these trials. None of the patients harboring either EGFR mutations or ALK fusions (0/15) achieved a major pathological response (MPR) in the LCMC3 study (2). This strongly suggested that these patients with driver genes unlikely benefited from neoadjuvant immunotherapy. Neoadjuvant targeted therapy might be more appropriate.

## Case presentation

**Figure 1:**
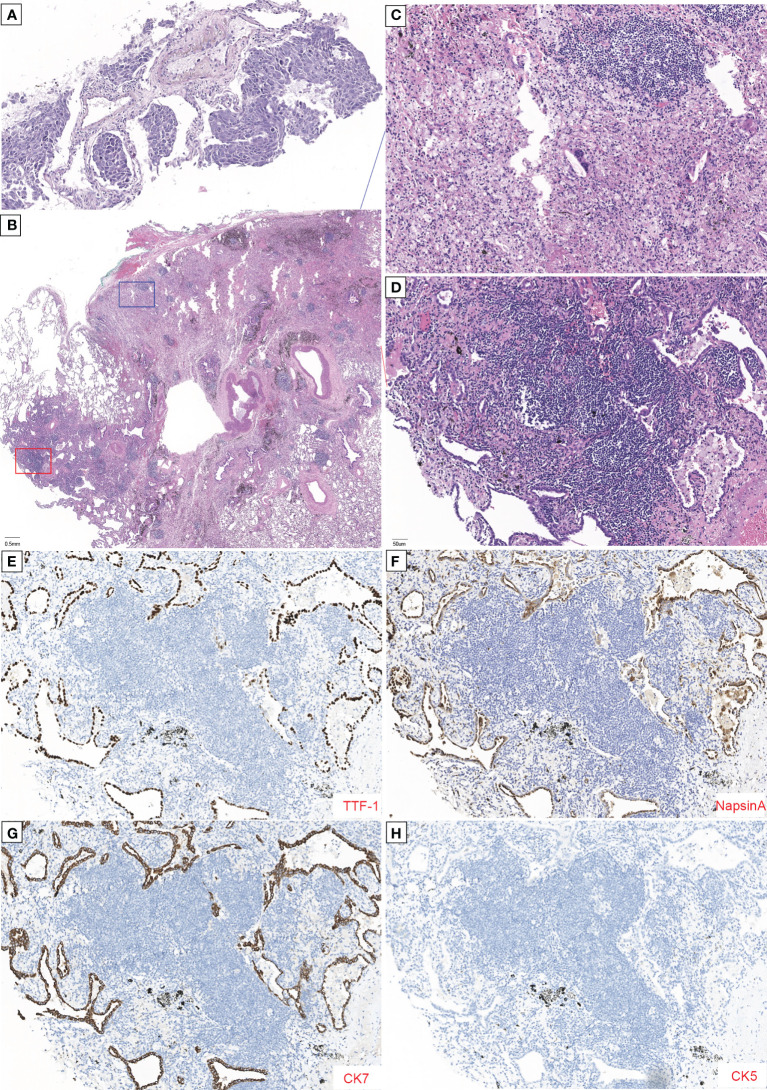
Histopathology of tumor with major pathological response (MPR) to neoadjuvant savolitinib. **(A)** High-power magnification (×200) shows tumor cell nests without inflammatory cell infiltration and initially diagnosed as a poorly differentiated carcinoma. **(B)** Low-power magnification (×20) shows that only 5% of this tumor was viable with 95% showing fibrosis and chronic inflammation without necrosis tissue in the pathology of postoperative samples by H&E staining. **(C, D)** Enlargement of the blue and red boxes in **(B)**. **(C)** H&E staining image shows numerous foam cells, multinucleated giant cells, and cholesterol crystals. No viable tumor was seen. **(D)** Higher power magnification shows the amount of inflammatory cell infiltration and the formation of lymphoid follicular, with the acinar type of tumor cells growing with adherence. Immunohistochemical (IHC) reveals that tumor cells were positive for TTF-1 **(E)**, Napsin A **(F)**, and CK7 **(G)** and negative for CK5 **(H)**. The scale length is 1 cm.

**Figure 2:**
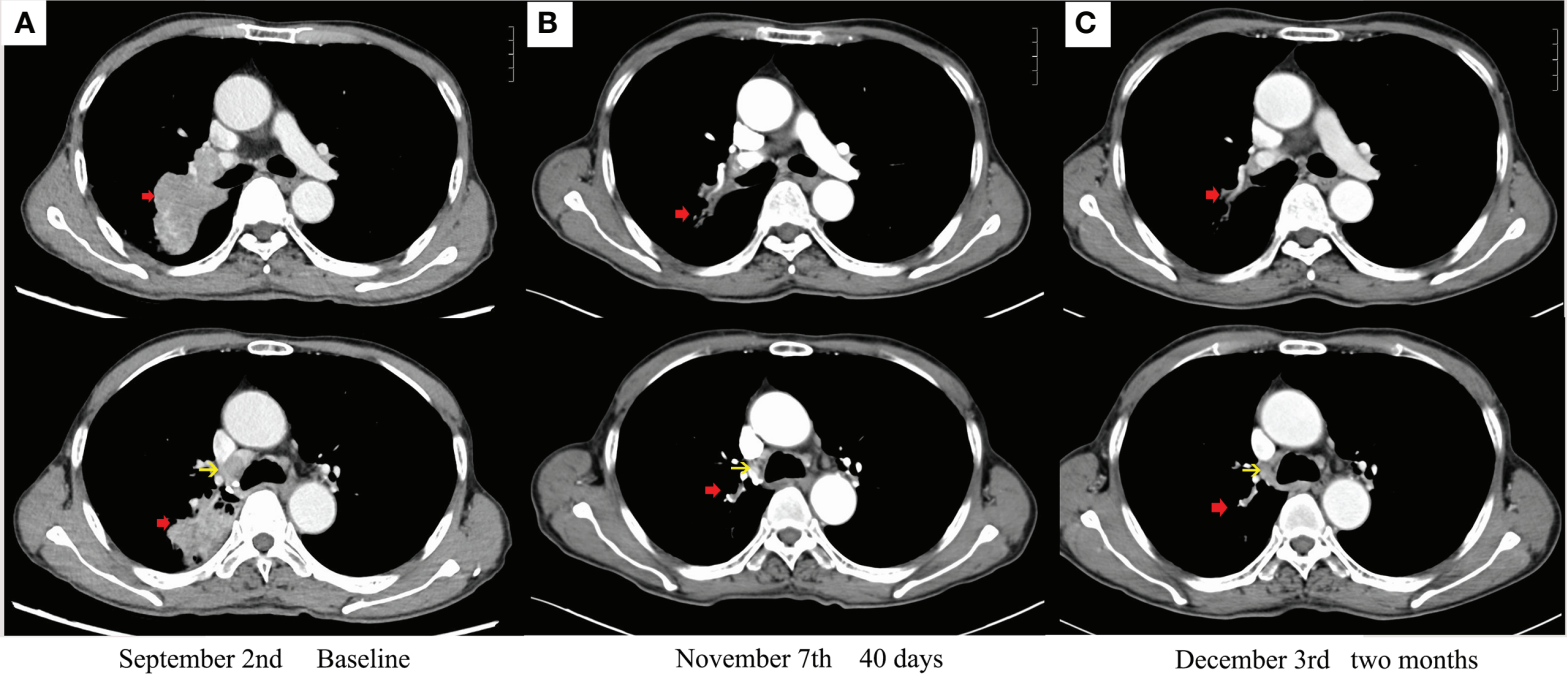
Enhanced chest CT scans during therapy: **(A)** baseline imaging. **(B)** After 40 days with savolitinib. **(C)** After 2 months with savolitinib. The red arrow indicates a primary tumor. The yellow arrow indicates metastatic lymph nodes.

A 76-year-old male patient with dyspnea, slight cough, weight loss (3 kg) in 2 months, and a history of smoking, without fever, chills, or syncope, visited our hospital on 7 September 2021. He had an Eastern Cooperative Oncology Group (ECOG) performance status of 2. A routine chest computed tomography (CT) scan showed a mass in the right lung, highly likely to be a tumor. A percutaneous pulmonary biopsy established the diagnosis of a poorly differentiated carcinoma (**Figure 1A**), with immunohistochemical (IHC) manifestation as CK (+), Ki-67 (~10%), TTF-1 (−), P63 (−), CgA (−), and Syn (−). A diagnostic workshop including enhanced CT scans of the chest and abdomen, MRI scan of the brain, and bone scintigraphy identified a soft tissue (5.8 × 4.9 cm) in the posterior segment of the right upper lobe, closely adjacent to the right superior lobe vein, compressing the bronchus, with right hilar and mediastinal lymph node enlargement (with a short diameter of 1.7 cm) (**Figure 2A**). Moreover, no distant metastasis was detected. His disease was evaluated as cT4N2M0, stage IIIB, which was considered a marginally resectable lesion through multidisciplinary team (MDT) discussion. Afterward, a next-generation sequencing (NGS) panel consisting of 56 driver genes (Burning Rock Biotech, China) was performed on his tumor sample and revealed MET exon 14 skipping mutation (METex14, 28.32%), point mutations in TP53, KDR, and KIT, and no EGFR/ALK alterations. PD-L1 expression and tumor mutation burden (TMB) were absent due to a limited biopsy sample. For unresectable locally advanced NSCLC, definitive chemoradiotherapy followed by durvalumab is the standard of care (SOC). Our patient was considered to have a marginally resectable disease through our MDT discussion, and then he was subjected to neoadjuvant therapy followed by surgical resection. The highly selective oral MET inhibitor savolitinib (HUTCHMED, AstraZeneca) was prescribed after getting the informed consent of the patient.

**Figure 3:**
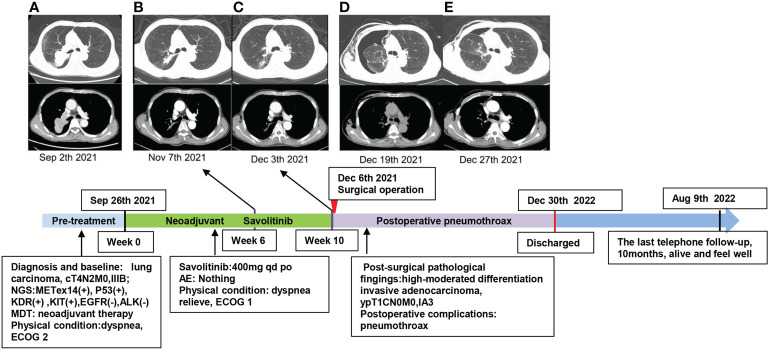
Timeline of the diagnosis and chest CT scans during the treatment and follow-up of this case. **(A)** Baseline imaging. **(B)** Treatment with savolitinib for 6 weeks. **(C)** Treatment with savolitinib for 10 weeks. **(D)** After 2 weeks of surgical operation, the patient had dyspnea, chest CT confirmed pneumothorax in the right lung, and closed thoracic drainage was performed on the patient. **(E)** Enhanced chest CT scans confirmed that the pneumothorax was improved 1 week later. The patient was alive and felt well at the last telephone follow-up on 9 August 2022.

Oral savolitinib was commenced at a dose of 400 mg once daily. Six weeks later, a CT rescan identified dramatic regressions of the primary tumor (shrinkage of 69%, **Figure 2B**) and lymph nodes. The tumor response was assessed as a partial response (PR). The treatment was well tolerated with no adverse events. The symptoms of dyspnea and cough were relieved and the ECOG PS returned to 1. Another CT scan after an additional 1 month indicated an even smaller tumor (shrinkage of 82%, **Figure 2C**). After our MDT consultation, surgery was recommended. The patient underwent video-assisted thoracic surgery (VATS). Right upper lobectomy, wedge resection for the dorsal segment of the right lower lobe, and mediastinal lymph node dissection were performed. During the operation, mild pleural adhesion was observed without pleural effusion or pleural implants, blood loss was 30 ml, and the operative time was 60 min. In the postsurgical pathological examination, all dissected lymph nodes including stations 2R (3), 4R (4), and 7 (5) were free of tumor cells. The primary tumor was identified as a highly–moderately differentiated invasive adenocarcinoma (**Figure 1B**), confirmed by IHC [positive for TTF-1 (**Figure 1E**), Napsin A (**Figure 1F**), and CK7 (**Figure 1G**) and negative for CK5/6 (**Figure 1H**)], with 5% of the residual viable tumor and 95% of fibrosis and inflammation (**Figures 1B–D**). MPR was achieved. The patient was discharged and followed up for 8 months. **Figure 3** demonstrates the timeline of the diagnosis, treatment, and follow-up of the patient.

## Discussion

Our patient with marginally resectable lung adenocarcinoma harboring METex14 was successfully treated with savolitinib as neoadjuvant therapy. Recently, neoadjuvant strategies have aroused great interest. A dozen of studies tested the efficiency of neoadjuvant therapies, including targeted therapy, immunotherapy, or immunochemotherapy (**Table 1**). In the LCMC3 study, the largest neoadjuvant immunotherapy trial, two cycles of preoperative atezolizumab led to an MPR of 20.4% (30/181) and a pCR of 6.8% (6). Neoadjuvant immunochemotherapy has higher efficiency. The MPR was typically 36.9%–85% and the pCR was 18%–38% except for a higher pCR of 63% from the NADIM study (7–11). In the CheckMate-816 study, the immunotherapy combination achieved a pCR of 24.0% and an MPR of 36.9%, compared with 2.2% and 8.9% for chemotherapy. The median event-free survival was 31.6 and 20.8 months, respectively (7). Regarding toxicities, the rate of grade 3 or worse adverse events could be as low as 16.6% (LCMC3) (6) or as high as 88% (SAKK16/14) (9). Notably, none of the patients harboring either EGFR mutations or ALK rearrangement (0/15) achieved MPR in the LCMC3 study (6). This strongly suggested that these patients were unlikely to benefit from neoadjuvant immunotherapy.

**Table 1:** Summary of the published prospective neoadjuvant therapy trials.

	Pts	Neoadjuvant (ICI+CTH; ICI; target)	MPR (%)	pCR (%)	Survival		≥Grade3 TRAE (%)
					mDFS	mOS	
Forde 2022 (7)	358	Nivo+CTH vs. CTH	36.9 vs. 8.9	24 vs. 2.2	31.6 vs. 20.8 months	Not reached	34 vs. 37
Rothschild 2021 (9)	55	Durv+CTH	62	18	12 months: 73.3%	Not reached (28.6 months+)	88
Provencio 2022 (8)	41	Nivo+CTH	83	63	3 years: 81.1% 4 years: 81.1%	3 years: 91.0% 42 months: 87.3%	30.4
Shu 2020 (10)	30	Atez+CTH	57	33	17.9 m	Not reached (27.6 months+)	50
Zinner 2020 (11)	13	Nivo+CTH	85	38	NR	NR	15.4
Carbone 2021 (6)	181	Atez	20.4	6.8	1 year: 85%	1 year: 92%	16.6
Cascone 2021 (12)	44	Nivo; Nivo+ipil	22; 38	10; 38	Not reached	Not reached (22.2 months+)	13;10
Zhang 2022 (13)	40	Sint	40.5	8.1	3 years: 75%	3 years: 88.5%	10
Wislez 2021 (14)	46	Durv	NR	NR	18 months: 69.7%	Not reached	0
Besse 2020 (15)	30	Atez	14	0	NR	NR	10
Forde 2018 (16)	21	Nivo	45	15	18 months: 73%	Not reached	4.5
Chao 2022 (17)	40	Osim	10.7	3.6	NR	NR	7.5
Zhong 2021 (18)	72	Erlo vs. CTH	9.7 vs. 0	0 vs. 0	21.5 vs. 11.4 months	42.2 vs. 36.9 months	0 vs. 29.4
Xiong 2020 (19)	15	TKI	67	0	NR	51.0 months	NR
Zhang 2021 (20)	33	Gefi	24.2	NR	33.5 months	Not reached	0
Xiong 2019 (21)	19	Erlo	NR	NR	11.2 months	51.6 months	15.8
Tan 2019 (22)	13	Gefi	7.7	NR	20.2 months	NR	8
Zhang 2019 (23)	11	Criz	NR	18.2	NR	NR	9

CHT, doublet chemotherapy; DFS, disease-free survival; MPR, major pathological response; OS, overall survival; pCR, complete pathologic response; Nivo, nivolumab; Durv, durvalumab; Atez, atezolizumab; Ipil, ipilimumab; Sint, sintilimab; Osim, osimertinib; Erlo, erlotinib; Gefi, gefitinib; Criz, crizotinib; NR, not reported.

For those with driver mutations, neoadjuvant targeted therapy might be more appropriate. In the EMERGING-CTONG1103 study, the only published RCT trial comparing neoadjuvant targeted therapy to platinum-based chemotherapy, neoadjuvant erlotinib achieved an MPR of 9.7% compared with 0% in the chemotherapy group. Moreover, the progression-free survival (PFS, 21.5 and 11.4 months) was significantly longer in the erlotinib group. Also, the overall survival (OS, 42.2 and 36.9 months) was numerically longer (18, 24). In another small-scale study, 11 patients with ALK rearrangement underwent surgery after neoadjuvant crizotinib therapy. Ten patients (91%) had R0 resection, including two cases of pCR (23). Furthermore, cases of successful neoadjuvant targeted therapy for ROS1, RET, or ALK rearrangement were reported (25–28). The prospective phase II ALNEO and NAUTIKA1 studies on neoadjuvant alectinib therapy finished enrolment, and preliminary results were reported (29, 30). For the completed resected patients harboring EGFR mutation, the phase III ADAURA study confirmed that osimertinib could achieve a longer DFS for stage IB to IIIA diseases. As a result, osimertinib is now recommended for these patients (31).

It should be noted that most, if not all, studies were performed on patients with resectable diseases. For those with marginally or potentially resectable lesions, preoperative therapy followed by surgery might also be possible. In an elegant pilot study, patients with initiative “unresectable” locally advanced lung cancer were successfully transformed into “resectable” disease by preoperative immunochemotherapy (32). This study highlighted the road to transformation of neoadjuvant treatment, given the high efficiency of the preoperative therapies.

METex14 is a low-frequency driver mutation in metastatic NSCLC (3%–4%) and is associated with a poor prognosis (1, 33). Patients with METex14 receiving chemotherapy had only an OS of 6.7 months (3). They also poorly responded to immune checkpoint inhibitors, with an objective response rate (ORR) of 17% and a PFS of 1.9 months (34). Non-specific inhibitors such as crizotinib brought an ORR of only 12% and a PFS of 2.6 months (35), which was far from satisfaction. Until now, three oral, highly selective, type Ib MET tyrosine kinase inhibitors (MET TKIs), namely savolitinib, tepotinib, and capmatinib, were approved for advanced NSCLC harboring METex14 (2, 4). Capmatinib and tepotinib were granted FDA approval, based on the results of the GEOMETRY mono-1 (capmatinib) and VISION (tepotinib) studies (4, 36, 37). In a crucial phase II trial conducted solely in a Chinese population (NCT02897479), savolitinib demonstrated an encouraging ORR of 49.2%, a disease control rate (DCR) of 93.4%, and a median overall survival (mOS) of 12.5 months (38). Savolitinib was approved for the treatment of advanced NSCLC patients with METex14 who progressed after or were intolerant to platinum-based chemotherapy (2). Savolitinib was the only one of this kind that got approval in China. Our patient was prescribed savolitinib.

Considering the high response rate of savolitinib, the neoadjuvant transformation strategy was explored in our patient. This treatment led to objective tumor regression, confirmed by the pathological response in the following radical surgery. In our case, we advocated the neoadjuvant MET TKI treatment for NSCLC. In support of our proposal, a phase 2 trial (NCT04926831) of perioperative capmatinib in NSCLC with METex14 or MET amplification is being conducted. The results of this trial are warranted.

## Data availability statement

The raw data supporting the conclusions of this article will be made available by the authors, without undue reservation.

## Ethics statement

Ethical review and approval was not required for the study on human participants in accordance with the local legislation and institutional requirements. The patients/participants provided their written informed consent to participate in this study. Written informed consent was obtained from the individual(s) for the publication of any potentially identifiable images or data included in this article.

## Author contributions

All authors conceived, directed, and supervised this paper. JT and ZL were responsible for data collection and manuscript writing. ZD revised the manuscript. All authors contributed to the article and approved the submitted version.

## Conflict of interest

The authors declare that the research was conducted in the absence of any commercial or financial relationships that could be construed as a potential conflict of interest.

## Publisher’s note

All claims expressed in this article are solely those of the authors and do not necessarily represent those of their affiliated organizations, or those of the publisher, the editors and the reviewers. Any product that may be evaluated in this article, or claim that may be made by its manufacturer, is not guaranteed or endorsed by the publisher.
